# Supplementary material for: Sustainability of effects and secondary long-term outcomes: One-year follow-up of a cluster-randomized controlled trial to prevent maltreatment in institutional care
Source: PLOS Glob Public Health. 2022 May 20;2(5):e0000286. doi: 10.1371/journal.pgph.0000286 (PMC10021849; doi:10.1371/journal.pgph.0000286)
Supplement: S1 Table — (DOCX) [file pgph.0000286.s001.docx]

**S1 Table:**

**Assessment of childcare knowledge**

In the last section of the questionnaire, we would like to know what you already know about childcare in general and in orphanages in particular. We have prepared a few questions to assess your knowledge. Please choose the write answers of the questions below. One or two answers can be correct for each of the questions. In the beginning, we would like to know how you would rate your own knowledge.

| 1. How would you rate your own knowledge about childcare? | [ 0 ] very little [ 1 ] some  [ 2 ] much [ 3 ] very much |
| --- | --- |
| 1. From what do children learn more? | [ a ] from what caregivers say  [ b ] from what caregivers do  [ c ] equally from what caregivers say and what they do |
| 1. Which strategy is helpful when a caregiver wants a child to stop beating another child? | [ a ] begging the child to stop  [ b ] promising a reward  [ c ] slapping the beating child  [ d ] tell the child to stop in direct and firm manner |
| 1. Which strategies help to maintain good behavior of children? | [ a ] praising the good behavior  [ b ] ignoring the good behavior  [ c ] rewarding the good behavior  [ d ] focusing on changing misbehavior |
| 1. Why is a good relationship between caregiver and child important? | [ a ] A good relationship between caregiver and child lays the base for a healthy development.  [ b ] With a good relationship between caregiver and child, the child will never show misbehavior.  [ c ] When the child respects the caregiver, the child will always behave well. |
| 1. How should caregivers behave to be a good role model? | [ a ] The caregiver should use the stick to punish misbehavior  [ b ] The caregiver should do what he/she says.  [ c ] The caregiver should make sure every child fears and respects him.  [ d ] The caregiver should show the behavior that they expect from the children. |
| 1. What is important when a caregiver wants to instruct a child? | [ a ] The caregiver needs to talk in very loud voice so that the child pays attention.  [ b ] The caregiver should make eye-contact with the child so that the child follows the instruction attentively.  [ c ] The instruction should be short and clear.  [ d ] The caregiver should explain the instruction with a lot of words so that the child really understands what to do. |
| 1. Which consequences does corporal punishment have? | [ a ] The child fears the person who uses corporal punishment.  [ b ] The child changes the behavior and behaves well.  [ c ] The child learns to solve conflicts with violence.  [ d ] The child learns to behave well in future. |
| 1. Why do children behave aggressively? | [ a ] They like to be aggressive and to molest others out of fun.  [ b ] They act aggressively because they experienced violence in the family origin and learned to solve conflicts with violence.  [ c ] They want to bother the caregiver.  [ d ] They are bad children. |

| 1. Which strategies may help a child to overcome bedwetting? | [ a ] Promising a reward for not bedwetting.  [ b ] Reminding the child to go to toilet before going to sleep.  [ c ] Paying attention that the child does not drink two hours before going to sleep.  [ d ] Instructing the child in front of the other children to teach him/her that bedwetting is embarrassing. |
| --- | --- |
| 1. Why do routines or rituals can help children to behave well? | [ a ] Routines and rituals help children to make their daily life predictable.  [ b ] Children should learn to follow rules.  [ c ] Routines and rituals help children to know what is expected from them in a certain situation.  [ d ] Without routines and rituals children are uncontrollable. |
| 1. Imagine the following situation: *One child is very angry, hits another child und takes away this toys.*   How should the caregiver react? | [ a ] The caregiver should punish the misbehaving child.  [ b ] The caregiver should shout at the misbehaving child.  [ c ] The caregiver should directly approach the children, firmly tell the misbehaving child to stop and separate them.  [ d ] The caregiver should beg the child to stop. |
