# Supplementary material for: Sustainability of effects and secondary long-term outcomes: One-year follow-up of a cluster-randomized controlled trial to prevent maltreatment in institutional care
Source: PLOS Glob Public Health. 2022 May 20;2(5):e0000286. doi: 10.1371/journal.pgph.0000286 (PMC10021849; doi:10.1371/journal.pgph.0000286)
Supplement: S2 Table — (DOCX) [file pgph.0000286.s002.docx]

S2 Table

Descriptive statistics of outcome variables

S2 Table
Descriptive statistics of outcome variables separated by intervention and waitlist orphanages

|  | Intervention | | | |  | Waitlist | | | | |
| --- | --- | --- | --- | --- | --- | --- | --- | --- | --- | --- |
|  | *n* | *M*  *(SD)* | *M_a_*  *(SD_a_)* | *M_ab_*  *(SD_ab_)* |  | *n* | *M*  *(SD)* | *M_a_*  *(SD_a_)* | *M_ab_*  *(SD_ab_)* | |
| **Reported levels of maltreatment** |  |  |  |  |  |  |  |  |  | |
| Reported levels of maltreatment (BA) | 81 | 27.72 (25.17) | 21.65  (9.89) | 0.00  (0.00) |  | 75 | 32.63  (38.90) | 20.38  (14.27) | 0.00  (0.00) | |
| Reported levels of maltreatment (FU 1) | 81 | 6.37  (9.85) | 3.41  (3.23) | 17.14  (10.54) |  | 75 | 16.21  (20.53) | 10.33  (6.29) | 5.80  (10.10) | |
| Reported levels of maltreatment (FU 2) | 81 | 5.47 (6.79) | 3.80  (2.46) | 16.73  (8.93) |  | 75 | 13.91  (21.15) | 7.27  (4.34) | 11.00  (12.94) | |
| **Attitudes towards violence** |  |  |  |  |  |  |  |  |  | |
| Attitudes towards violence (BA) | 81 | 4.10  (3.25) | 3.61  (1.37) | 0.00  (0.00) |  | 75 | 3.75  (3.59) | 3.11  (1.54) | 0.00  (0.00) | |
| Attitudes towards violence (FU 1) | 81 | 1.27  (1.86) | 0.65  (0.83) | 2.86  (1.41) |  | 75 | 2.92  (2.57) | 2.53  (1.39) | 0.33  (0.71) | |
| Attitudes towards violence (FU 2) | 81 | 1.06  (1.34) | 0.71  (0.76) | 2.55  (1.34) |  | 75 | 1.96  (2.05) | 1.53  (0.87) | 1.53  (1.52) | |
| **Childcare knowledge** |  |  |  |  |  |  |  |  |  | |
| Childcare knowledge  (BA) | 81 | 29.90  (4.39) | 30.39  (2.13) | 0.00  (0.00) |  | 75 | 30.32  (3.39) | 30.58  (1.51) | 0.00  (0.00) | |
| Childcare knowledge  (FU 1) | 81 | 33.23  (3.49) | 33.43  (1.90) | -3.16  (1.87) |  | 75 | 30.56  (3.93) | 30.89  (2.04) | -0.04  (0.47) | |
| Childcare knowledge  (FU 2) | 81 | 33.25  (3.17) | 33.37  (1.39) | -3.18  (2.20) |  | 75 | 33.53  (2.98) | 33.71  (1.29) | -2.98  (1.48) | |
| **Caregiver-child relationship** |  |  |  |  |  |  |  |  |  | |
| Caregiver-child relationship (BA) | 81 | 10.22  (3.39) | 9.96  (1.71) | 0.00  (0.00) |  | 75 | 10.27  (3.26) | 9.84  (1.59) | 0.00  (0.00) | |
| Caregiver-child relationship (FU 1) | 81 | 9.43  (3.51) | 8.96  (1.73) | 0.90  (1.56) |  | 75 | 9.72  (2.77) | 9.38  (1.45) | 0.16  (0.52) | |
| Caregiver-child relationship (FU 2) | 81 | 8.62  (2.73) | 8.41  (1.44) | 1.49  (1.49) |  | 75 | 9.57  (3.13) | 9.44  (1.65) | 0.89  (1.53) | |
| **Stress level** |  |  |  |  |  |  |  |  |  | |
| Stress level  (BA) | 81 | 22.43  (15.70) | 19.66  (6.82) | 0.00  (0.00) |  | 75 | 21.73  (17.32) | 18.14  (7.95) | 0.00  (0.00) | |
| Stress level  (FU 1) | 81 | 18.62  (17.96) | 14.26  (9.91) | 4.33  (6.07) |  | 75 | 17.79  (13.93) | 15.31  (7.22) | 1.40  (2.94) | |
| Stress level  (FU 2) | 81 | 13.10  (12.76) | 10.44  (4.08) | 8.25  (6.24) |  | 75 | 17.63  (13.59) | 15.19  (6.05) | 4.06  (6.95) | |
| **Mental problems** |  |  |  |  |  |  |  |  |  | |
| mental problems (BA) | 81 | 7.07  (9.09) | 4.37  (3.66) | 0.00  (0.00) |  | 75 | 7.68  (8.83) | 5.16  (3.67) | 0.00  (0.00) | |
| mental problems  (FU 1) | 81 | 5.75  (7.80) | 3.29  (3.16) | 0.47  (1.92) |  | 75 | 5.23  (7.15) | 3.13  (1.97) | 0.62  (1.25) | |
| mental problems  (FU 2) | 81 | 2.73  (4.64) | 1.22  (1.31) | 2.53  (2.78) |  | 75 | 4.75  (5.36) | 3.27  (1.71) | 1.69  (3.18) | |
| *Note. n =* number of respondents, *M =* mean, *SD =* standard deviation. a = based on 20% trimmed means, b = based on transformed values. BA = baseline, FU 1 = first follow-up, FU 2 = second follow-up | | | | | | | | | |  |
