# Supplementary material for: Sustainability of effects and secondary long-term outcomes: One-year follow-up of a cluster-randomized controlled trial to prevent maltreatment in institutional care
Source: PLOS Glob Public Health. 2022 May 20;2(5):e0000286. doi: 10.1371/journal.pgph.0000286 (PMC10021849; doi:10.1371/journal.pgph.0000286)
Supplement: Attachment — Submitted filename: Rebuttal_letter.docx [file pgph.0000286.s004.docx]

Dear Dr Heine,

Thank you and the reviewers again for your time and comments on our manuscript (PGPH-D-21-00959) entitled ***“Sustainability of effects and secondary long-term outcomes: One-year follow-up of a cluster-randomized controlled trial to prevent maltreatment in institutional care”.*** We are happy about the positive feedback as well as the appreciation and acknowledgement of the efforts we made to conduct research under these challenging conditions. Thank you for your willingness to consider a revised version of our manuscript. The reviewer comments and journal requirements were very straightforward to implement. In what follows, we describe how we addressed each of the points that the reviewers raised.

**Journal Requirements:**

- Please update the completed 'Competing Interests' statement.

***Response:*** We have updated the 'Competing Interests' statement.

- Please provide a complete Data Availability Statement in the submission form, ensuring you include all necessary access information or a reason for why you are unable to make your data freely accessible.

***Response:*** We have updated the Data Availability Statement and provide a dataset as supporting information file.

- 3. Please provide separate figure files in .tif or .eps format only and ensure that all files are under our size limit of 20MB.

***Response:*** We have revised the figures accordingly.

- Please include a legend for Figure 1 in your manuscript.

***Response:*** We have now included a legend for Figure 1 in our manuscript.

- Thank you for providing an Ethics Statement for your study. Currently, your ethics statement appears outside of your Methods section of  your manuscript (introduction, results, conclusions, acknowledgements). Please ensure that the Ethics statement appears only in your Methods section and remove any other occurances.

***Response:*** We have now included the Ethics statements in the Methods section.

- We have noticed that you have uploaded supporting information but you have not included a list of legends.  Please add a full list of legends for all supporting information files (including figures, table and data files) after the references list.

***Response:*** We have added a list of legends of our supporting information files after the references list.

- Please review your reference list to ensure that it is complete and correct. Any changes to the reference list should be mentioned in the rebuttal letter that accompanies your revised manuscript.

***Response:*** We apologize for the inconsistencies in our in-text citations and the references list. We have overlocked these inconsistencies after changing the citation style using our software. We have now resolved all these issues.

**Reviewer #1:**

- Mixed referencing styles - at times you give authors and date, and at other times you list numerical numbers)

***Response:*** Thanks for the hint. See our response to the last comment of the journal requirements.

- In your introduction you justify orphanages. Perhaps given the enormity of the problems you could give a stronger statement of how this is a negative environment and how despite global guidance orphanages persist (rather than your bland stataement that deinstitutionalized forms of care (perhaps name it as family based care) is not provided!! (Who says it cannot be?)

***Response:*** We agree with the reviewer and have revised the first paragraph (p. 3) as follows:

*“In 2015, the number of orphans globally was estimated to be 140 million (1), the majority of which living in Sub-Saharan Africa (2). Besides loss due to AIDS/HIV or war, other parental illnesses, poverty, disabilities, and abandonment result in children growing up without their parents (3,4). Though family-based care generally promotes the best outcomes for children (5), current challenges in developing countries, such as the lack of financial resources, governmental support, and professional social-work infrastructure as well as an increasing number of children in need (4), result in the lack of family-based care settings (6) and the persistence of institutional care facilities short and medium term. In addition to advocacy for and introduction of family-based care, there is also a need for approaches which emphasize the improvement of institutional care (7).”*

- The essential limitation is that the responses are all self report with no validation by the child or even an observer. The possibility of response bias is high and it may well be that the caregivers pick up the social acceptability during the course and respond but there may be a vast gap between attitudes and behaviour. This is a fundamental weakness of this intervention evaluation and needs to be raised and discussed in the discussion.

***Response:*** We must acknowledge this weakness of our otherwise carefully conducted study. We have added this as a limitation to the limitation section (p. 20) as follows:

*“Caregivers were not blinded, and social desirability cannot be ruled out as we assessed only self-report data. Objective assessments like observations are needed to address this problem. Moreover, the findings reported here relate exclusively to caregivers' self-reports. Additional reports from the affected children themselves would have been less biased and would be essential to include in future studies.”*

- Discussion. The authors need to insert "reported levels" into all their findings. We do not know if there was reduced levels of maltreatment - only reduced reported levels of maltreatment.

***Response:*** We thank the reviewer pointing us to this inaccuracy. We have changed this accordingly throughout the manuscript.

- It is a worthy finding to see that changed attitudes persisted over time. It would be important to add some reflections on how this may translate into behaviour and perhaps even how a future study may be set up to measure behaviour rather than confined to self reported attitiudes.

***Response:*** We thank the reviewer for this insight. We have included the suggested ideas in the discussion section (p. 19) as follows:

*“It is particularly noteworthy that changed attitudes persisted over time as the presence of positive attitudes toward violent discipline has been identified as a robust predictor of violent behavior (44). The fact that we were able to show enduring training effects using the two-week ICC-C training workshop alone indicates that even brief intervention approaches can initiate long-term changes. Nevertheless, it is of central importance to examine whether the self-reported behavioral changes are reflected in changed behavior. In the future, it will be important to include more objective observational measures and the reports of the affected children in the evaluation of ICC-C and comparable interventions.”*

- It would be crucial that the authors add a paragraph to the discussion on how intolerable such levels of violence are and that trainng is just a small intervention in a crisis, but a higher level longer term child protection lens may be vital to totally reassess this form of care.

***Response:*** We agree with the reviewer that caregiver training is only one piece of the puzzle and not the end of the story. We have therefore added a sentence on this in the conclusions section (p. 22). It reads as follows:

*“Nevertheless, we must note that training for caregivers can only be one piece of the puzzle that protects children in institutional care from violence and neglect in the short and medium term. The goal of child protection efforts should be the establishment of high-quality family-based care, which should eventually make institutional care unnecessary (5).”*

**Reviewer #2:**

- It seems like the secondary outcomes were significant than the primary ones. Could the authors shed some light on why that was the case?

***Response:*** We share the observation that the effects related to attitudes towards violence and childcare knowledge, especially in the waiting list group are stronger than the reported level of maltreatment. Knowledge and attitudes are prerequisites for behavior change. The finding is therefore not that surprising. Knowledge and attitudes are prerequisites for behavior change, but they do not necessarily lead to behavior change in every situation. We have now tried to make this clearer in the discussion, for example by adding the following information:

*“It is particularly noteworthy that changed attitudes persisted over time as the presence of positive attitudes toward violent discipline has been identified as a robust predictor of violent behavior (45). The fact that we were able to show enduring training effects using the two-week ICC-C training workshop alone indicates that even brief intervention approaches can initiate long-term changes. Nevertheless, it is of central importance to examine whether the self-reported behavioral changes are reflected in changed behavior. In the future, it will be important to include more objective observational measures and the reports of the affected children in the evaluation of ICC-C and comparable interventions.”*

*“Providing information can be a strong predictor for developing skills (43) and knowledge is an important precondition of behavior and a determinant for behavioral change (44). The improved knowledge provides a base for heightening the quality of childcare in orphanages.”*

- Structural factors may play a key influence on outcomes within orphanages/institutional care settings and may in some cases interfere with a well designed and implemented intervention. In as much as this was not the focus of this paper, do the authors have a sense of the role of structural factors on their intervention?

***Response:*** Undoubtedly, structural factors may play a role in terms of effectiveness, however, the orphanages in our study were so diverse that we could hardly form and analyze subgroups. Analysis at the level of individual orphanages is difficult due to the low number of cases. However, it was also not our main aim in this study to test which factors make success more likely or less likely. Rather, the goal was to show that the intervention is effective under real-world conditions in very heterogenous settings. However, a future step could very well be to identify structural factors. Therefore, we have added the following sentence to the limitation sections (p. 21):

*“Structural factors in individual orphanages, such as such as material and personnel equipment, type of sponsorship, etc., could have influenced effects. Therefore, it would be interesting to determine and consider the influence of possible structural factors in the future.”*

- Were there any differences in outcomes by institution type? The authors mention that the orphanages/ institutional care facilities were heterogeneous. What exactly do they mean by this? What are some of the differences that were clear within the orphanages and among caregivers that readers need to be aware off in the interpretation of these findings?

***Response:*** To explain further what we mean by the orphanages being heterogenous, we have added some more information on the orphanages to the methods section (p. 6). The added information reads as follows:

*“****Orphanages.*** *Overall, the orphanages were very heterogeneous in terms of material and human resources, sponsors (public, religious, community-based), professionalism of management, number of caregivers and children. However, it reflects the reality of life for orphans in Dar es Salaam. Out of the 24 institutions five orphanages were fully registered, 10 were in the process of registration and 9 orphanages were not registered. Between 13 - 121 children and adolescents (Mdn = 46), ranging from 0 to 28 years of age (note that some young adults are supported for studies at university) were living in the orphanages. The average caregiver-child ratio was 1:10, range 1:5 – 1:23.”*

However, as we have explained in our previous response, we have not yet analyzed the differences between orphanages in terms of outcomes.

- The authors need to rework the references throughout the paper. There seems to be more than one referencing style used in the paper. Some references cited in the paper are also missing in the reference list.

***Response:*** Thanks for the hint. See our response to the last comment of the journal requirements.

We believe that with the changes made to the manuscript we have sufficiently addressed the remaining concerns brought up by the reviewers. We hope that you now find the manuscript suitable for publishing. Thank you again for your time and consideration. We look forward to hearing from you.

Kind regards,

Tobias Hecker

Corresponding Author
