# Supplementary material for: Sustainability of effects and secondary long-term outcomes: One-year follow-up of a cluster-randomized controlled trial to prevent maltreatment in institutional care
Source: PLOS Glob Public Health. 2022 May 20;2(5):e0000286. doi: 10.1371/journal.pgph.0000286 (PMC10021849; doi:10.1371/journal.pgph.0000286)
Supplement: Attachment — Submitted filename: Rebuttal_letter_2nd revision.docx [file pgph.0000286.s005.docx]

Dear Dr Heine,

Thank you and the additional reviewers again for your time and comments on our manuscript (PGPH-D-21-00959) entitled ***“Sustainability of effects and secondary long-term outcomes: One-year follow-up of a cluster-randomized controlled trial to prevent maltreatment in institutional care”.*** We are happy about the positive feedback as well as the appreciation and acknowledgement of the efforts we made to conduct research under these challenging conditions. The reviewer comments and journal requirements were very straightforward to implement. In what follows, we describe how we addressed each of the points that the reviewers raised.

**Journal Requirements:**

- Please update the completed 'Competing Interests' statement.

***Response:*** We have updated the 'Competing Interests' statement.

- Please review your reference list to ensure that it is complete and correct. Any changes to the reference list should be mentioned in the rebuttal letter that accompanies your revised manuscript.

***Response:*** We have reviewed our reference list to ensure that it is complete and correct.

**Reviewer #3:**

- Page 4, bottom. Ref 9 showed better effects after 9 than 4 months of the intervention.

***Response:*** We thank the reviewer for this additional hint. We have added this information on p. 4 as follows:

*“Trainings like these also showed additional positive effects on caregivers’ stress and mental health: After receiving an intervention, the caregivers reported reduced job stress, anxiety, and depression (9). The effects were stronger nine months after the intervention compared to four months after the intervention.”*

- Page 7, Table 1. Were there any significant differences between the intervention and Waitlist group on these characteristics that might explain any differences between these groups?

***Response:*** Indeed, this question is not so easily answered. This is due to the heterogeneity of the institutional care facilities. The differences within the groups were greater than between the groups. We have taken this into account in our analyses. However, we cannot easily compare the groups in the descriptive statistics. We have now made this clear again in the table notes as follows:

*“Because of cluster effects no inference statistical analysis is made.”*

- Page 17. I wonder if the premature changes in the waitlist group could be because of familiarity with the assessments that tell them what you are looking for and value?

***Response:*** We thank the reviewer for this idea. This might indeed be possible. We have integrated this idea in p. 17 as follows:

*“Another explanation could also be that participants in the waiting list group were also already familiar with the assessment tools in the follow-up interviews and were thus more likely to be concerned about positive self-presentation.”*

We believe that with the changes made to the manuscript we have sufficiently addressed the remaining concerns brought up by the additional reviewers. We hope that you now find the manuscript suitable for publishing. Thank you again for your time and consideration. We look forward to hearing from you.

Kind regards,

Tobias Hecker

Corresponding Author
